# What Do We Know about Transcranial Direct Current Stimulation for Major Depression?

**DOI:** 10.3390/brainsci10080480

**Published:** 2020-07-25

**Authors:** Derrick Matthew Buchanan, Philippe Robaey, Amedeo D’Angiulli

**Affiliations:** 1 Department of Neuroscience, Carleton University, Ottawa, ON K1S 5B6, Canada; MatthewBuchanan@cmail.carleton.ca (D.M.B.); PRobaey@cheo.on.ca (P.R.); 2 Neuroscience of Imagination Cognition Emotion Research Lab, Carleton University, Ottawa, ON K1S 5B6, Canada; 3 Neuropsychiatry Lab, Children’s Hospital of Eastern Ontario Research Institute, Ottawa, ON K1H 8L1, Canada; 4 Department of Psychiatry, University of Ottawa, Ottawa, ON K1N 6N5, Canada

**Keywords:** transcranial direct current stimulation, tDCS, non-invasive brain stimulation, NIBS, transcranial magnetic stimulation, transcranial magnetic stimulation (TMS), major depression

## Abstract

The interest in using non-invasive brain stimulation (NIBS) for the treatment of major depression (MD), including treatment resistant depression, is growing rapidly. The paper by Bennabi and Haffen (*Brain Sci.* **2018**, *8*) was an important step towards the formal acceptance of transcranial direct current stimulation (tDCS) as a possible form of therapy. Their review demonstrated favourable support for the beneficial effects of tDCS for MD, coupled with necessary practical considerations, such as its relatively low cost, portability/ease of use in clinical settings, non-invasiveness, and good tolerability. Here, we provide a follow-up to their review and sketch a current update. Means for optimizing tDCS efficacy and potential limitations of current studies are discussed.

The interest in using non-invasive brain stimulation (NIBS) for the treatment of major depression (MD), including treatment resistant depression, is growing rapidly. The paper by Bennabi and Haffen [1] contributed an important assessment for the formal acceptance of transcranial direct current stimulation (tDCS) as a possible form of therapy. tDCS is a NIBS technique which consists of applying a weak electric current (1–2 mA) to the patient’s scalp via two or more electrodes positioned in correspondence with cortical regions of interest. Bennabi and Haffen’s review demonstrated favorable support for the beneficial effects of tDCS for MD (effect size 0.35) but weak support for treatment resistant depression. This was coupled with a report on the necessary practical considerations, such as its relatively low cost, portability/ease of use in clinical settings, non-invasiveness, and good tolerability. With the widespread uptake and now largely regulated transcranial magnetic stimulation (TMS) for MD, it seems only logical that the regulatory induction of tDCS may be the next step. At the moment, tDCS has a level B recommendation for treating MD in the EU [1,2]. Here, we provide a follow-up to Bennabi and Haffen [1] and sketch a current update.

While the efficacy of tDCS is being investigated in a broad array of psychiatric and neurological conditions [3,4], its potential in major depression is among the most promising [1]. Boggio et al. [5] and others [6] have presented strong evidence for tDCS efficacy in major depression, with a significant decrease in depressive symptoms ranging from 24.8–40.4% in active conditions compared to 10.4–15.9% in sham. Unfortunately, in the decade since these early clinical trials, the results have not been straightforward. According to recent meta-analyses, tDCS used as monotherapy was associated with significantly higher response rates when compared to sham tDCS [7,8], and active tDCS was superior to sham regarding endpoint depression scores and achieved a superior response and rates [8]. However, in multiple studies in which the possible effects of placebo or other concurrent treatments were properly controlled, reductions in depression symptoms often were non-significant when compared to sham. This is further evidenced by an international, randomized, controlled trial of 130 participants which found no antidepressant difference between active and sham tDCS [9]. In addition to the possibility of treatment resistant patients skewing data, there are a few leading explanations for these discrepancies in the literature.

One of the current hypotheses in the tDCS literature [10,11,12], which is concurrent with TMS literature [13], is that tDCS dose (i.e., current intensity, session duration, and number of sessions), electrode montage, and other stimulation parameters may need to be individualized in order to optimize efficacy. This is further based on computational magnetic resonance imaging (MRI) evidence [14,15] that demonstrated how individual brain and skull morphology/density can influence current flow. Therefore, the efficacy of studies with a singular electrode location and static amperage for all subjects may be limited. To mitigate this, it is proposed that the current flow should be optimized by using MRI or electroencephalography (EEG) [16]. This ensures that the optimal amount of current is reaching the region of interest for each individual subject. This is akin to how TMS clinics optimize dose response curves by locating the primary motor cortex (M1) hotspot by measuring the threshold of the motor evoked response (MEP).

Another potential explanation for the inconsistent results of tDCS for MD is the various subtypes and endophenotypes that have been identified for MD [17,18]. In 2017, Drysdale et al. [17] published an article which identified four unique dysfunctional neurophysiological subtypes of MD. This was based on a multi-site sample of 1188 subjects who were also assessed with functional MRI. Connectivity analyses differentiated patients by limbic and frontostriatal networks into four biotypes with 82–93% sensitivity and specificity. The same study further demonstrated in a subsample of 154 subjects that these biotypes also predicted responsiveness to TMS. Therefore, tailoring tDCS protocols to these subtypes and other individualized biomarkers should similarly yield better outcomes.

Although current evidence suggests weak antidepressant effects of tDCS in treatment resistant groups [1] it still remains a hopeful avenue [19]. Patients with treatment resistant depression will try at least two to four medications before finding one that is right for them or finding out that they are resistant to all of them. Given that the effects of medication can take weeks to manifest, this process is a major burden on the patient. Moreover, pharmaceuticals come with a long list of undesirable side effects. It is crucial to provide these patients with alternative treatment options that may be more suitable to their needs. In a study comparing the acceptability of tDCS to pharmaceuticals, subjects were consistently partial to tDCS [20]. Interestingly, this is also the case in children [20]. Therefore, the community is prepared to embrace the uptake of tDCS if its efficacy is established. The onus is on the researchers and clinicians to advance the optimization of tDCS in order to increase its efficacy.

In general, the non-invasive nature of tDCS and its overall lack of side effects make it an appealing option for treating children and adolescents for the same conditions as considered in adults [21]. For parents, tDCS is a treatment option that offers hope for a short-term, intensive treatment option, compared to the prospect of years of being medicated [20]. That being said, tDCS studies in children are mostly limited to treating neurodevelopmental conditions like attention deficit hyperactivity disorder (ADHD), autism, or cerebral palsy (CP) [4]. tDCS studies on pediatric depression are scarce [22]. There have, however, been a handful of open label TMS studies that have demonstrated encouraging evidence for treating pediatric depression, including treatment resistant depression, and suicidality [23,24].

It is also well known that medication use can influence cortical excitability and that this may interact with the effects of tDCS [25]. Therefore, it is possible that medication may either facilitate or interfere with the antidepressant effects of tDCS. It is likely that either facilitation or interference could occur depending on the other stimulation parameters and individual differences. Studies should carefully control for this, and special consideration should be made for tDCS protocols including concomitant medication.

Finally, use of NIBS is not restricted to clinical settings. It may also be more convenient for patients to receive their treatment remotely from the comfort of their home. Indeed, several treatment studies [26,27] and multiple guidelines [28,29] for remotely supervised and at-home tDCS have been published. In a recent pilot trial of remotely supervised tDCS for major depression, patients saw a significant improvement from baseline up to one month after their treatment ended [30]. At home treatments are particularly important in the current climate of COVID-19. A recent guideline was published by Bikson et al. [31] to outline the necessity and importance of continuing NIBS treatments and the steps to doing this safely during the pandemic.

In conclusion, there still appears to be a split in the literature regarding the efficacy of tDCS for treating major depression. It is possible that the heterogeneity of results is due to unspecific tDCS protocols. Such as with TMS, it is expected that the optimization of tDCS protocols by consideration of disease subtype, endophenotype, and individual neuroimaging should increase the efficacy of tDCS.
